# Genetic Mutation Analysis of Human Gastric Adenocarcinomas Using Ion Torrent Sequencing Platform

**DOI:** 10.1371/journal.pone.0100442

**Published:** 2014-07-15

**Authors:** Zhi Xu, Xinying Huo, Hua Ye, Chuanning Tang, Vijayalakshmi Nandakumar, Feng Lou, Dandan Zhang, Haichao Dong, Hong Sun, Shouwen Jiang, Guangchun Zhang, Zhiyuan Liu, Zhishou Dong, Baishuai Guo, Yan He, Chaowei Yan, Lu Wang, Ziyi Su, Yangyang Li, Dongying Gu, Xiaojing Zhang, Xiaomin Wu, Xiaowei Wei, Lingzhi Hong, Yangmei Zhang, Jinsong Yang, Yonglin Gong, Cuiju Tang, Lindsey Jones, Xue F. Huang, Si-Yi Chen, Jinfei Chen

**Affiliations:** 1 Department of Oncology, The Affiliated Nanjing First Hospital, Nanjing Medical University, Nanjing, China; 2 San Valley Biotechnology Incorporated, Beijing, China; 3 Norris Comprehensive Cancer Center, Department of Molecular Microbiology and Immunology, Keck School of Medicine, University of Southern California, Los Angeles, California, United States of America; National Cancer Center, Japan

## Abstract

Gastric cancer is the one of the major causes of cancer-related death, especially in Asia. Gastric adenocarcinoma, the most common type of gastric cancer, is heterogeneous and its incidence and cause varies widely with geographical regions, gender, ethnicity, and diet. Since unique mutations have been observed in individual human cancer samples, identification and characterization of the molecular alterations underlying individual gastric adenocarcinomas is a critical step for developing more effective, personalized therapies. Until recently, identifying genetic mutations on an individual basis by DNA sequencing remained a daunting task. Recent advances in new next-generation DNA sequencing technologies, such as the semiconductor-based Ion Torrent sequencing platform, makes DNA sequencing cheaper, faster, and more reliable. In this study, we aim to identify genetic mutations in the genes which are targeted by drugs in clinical use or are under development in individual human gastric adenocarcinoma samples using Ion Torrent sequencing. We sequenced 737 loci from 45 cancer-related genes in 238 human gastric adenocarcinoma samples using the Ion Torrent Ampliseq Cancer Panel. The sequencing analysis revealed a high occurrence of mutations along the TP53 locus (9.7%) in our sample set. Thus, this study indicates the utility of a cost and time efficient tool such as Ion Torrent sequencing to screen cancer mutations for the development of personalized cancer therapy.

## Introduction

Gastric cancer is the second most common cancer worldwide with a frequency that varies greatly across different geographic locations. Its incidence is highest in Japan, Eastern Asia, South America, and Eastern Europe, whereas Canada, Northern Europe, Africa, and United States have the lowest incidences [1]. However, it remains the third most common gastrointestinal malignancy in North America after colorectal and pancreatic cancer and commonly occurs after 40 years of age [2]. The Lauren classification divides gastric cancer into two major histologic types: intestinal or diffuse. Diffuse-type cancers have noncohesive tumor cells diffusely infiltrating the stroma of the stomach and often exhibit deep infiltration of the stomach wall with little or no gland formation. Intestinal-type cancers, on the other hand, show recognizable gland formation similar in microscopic appearance to colonic mucosa [3]. Most gastric cancers are sporadic but 8–10% are genetically inherited [2].

Many commonly activated oncogenes have been shown to harbor mutations in gastric cancer. Single or combinatorial therapeutics targeting genetic mutations is becoming attractive options in the treatment of gastric cancers. For example, trastuzumab was approved in combination with chemotherapy for the treatment of ERBB2-positive gastric cancers [4]. EGFR, another receptor tyrosine kinase is noted for its overexpression in some gastric cancers and trials employing the use of EGFR inhibitors are currently underway [5]. Similarly, gastric cancers are associated with the overexpression or amplification of other molecules such as MET, MSTIR, and FGFR2, and multiple trails testing the efficacy of inhibitors against these molecular mutations are also ongoing [6], [7].

Despite several improvements made in treating and screening for gastric cancers, the prognosis of patients with gastric adenocarcinoma remains poor [8]. To understand and develop new therapeutics and treat patients with gastric adenocarcinoma more effectively, it is essential to profile the individual cancer genome and dissect the oncogenic mechanisms that regulate the progression of gastric cancer, which may form the foundation for individualized, tailored therapy. Next-generation sequencing technologies have revolutionized cancer genomics research by providing an unbiased and comprehensive method of detecting somatic cancer genome alterations [9]. These technologies have several advantages over Sanger sequencing by capillary electrophoresis such as the ability to sequence gigabases of nucleotides to detect genetic mosaicism in depth [10]. However, routine usage of these technologies leaves us with several limitations such as the cost of entry, long processing time, and sample scalability. Recently, a new Ion Torrent sequencing technology based on semiconductor sequencing [11] has substantially circumvented many of these issues. The Ion Torrent method relies on standard DNA polymerase sequencing with unmodified dNTPs but uses semiconductor-based detection of hydrogen ions released during every cycle of DNA polymerization [11]. Each nucleotide incorporation into the growing complementary DNA strand causes the release of a hydrogen ion that is sensed by a hypersensitive ion sensor [11]. Ion Torrent Personal Genome Machine (PGM) can currently generate 10 Mb pairs (Mbp) of sequence data on the first-generation 314 chip within several hours of machine run time. In this study, we have sequenced 238 clinical gastric adenocarcinoma samples to identify genetic mutations in 737 loci of 45 cancer-related genes.

## Materials and Methods

### Ethics statement

The study has been approved by the Ethical Committee of the Affiliated Nanjing First Hospital, Nanjing Medical University, China. For formalin fixed and paraffin embedded (FFPE) tumor samples, no informed consent was available, therefore all samples and medical data used in this study have been irreversibly anonymized.

### Patient information

Tumor samples used in the study were collected from the Affiliated Nanjing First Hospital, Nanjing Medical University, China. A total of 238 FFPE tumor samples from gastric adenocarcinoma patients were analyzed. The mean age of 238 patients was 60 years (range, 28–81). Out of 238 samples, 135 tumors were diffuse and 103 tumors were intestinal.

### DNA preparation

DNA was isolated from FFPE samples after deparaffinization and extraction of 3–5 µm thick paraffin sections in xylene and by using the QIAamp DNA Mini Kit (Qiagen) per the manufacturer's instructions.

### Ion Torrent PGM Library Preparation and Sequencing

An Ion Torrent adapter-ligated library was made following the manufacturer's Ion AmpliSeq Library Kit 2.0 protocol (Life Technologies, Part #4475345 Rev. A). Briefly, 50 ng pooled amplicons were end-repaired, and Ion Torrent adapters P1 and A were ligated using DNA ligase. Following AMPure bead (Beckman Coulter, Brea, CA, USA) purification, adapter-ligated products were nick-translated and PCR-amplified for a total of 10 cycles. The resulting library was purified using AMPure beads (Beckman Coulter) and the concentration and size of the library determined using the Agilent 2100 Bioanalyzer (Agilent Technologies) and Agilent BioAnalyzer DNA High-Sensitivity LabChip (Agilent Technologies).

Sample emulsion PCR, emulsion breaking, and enrichment were performed using the Ion Xpress Template Kit (Part #4467389 Rev. B), according to the manufacturer's instructions. Briefly, an input concentration of one DNA template copy/Ion Sphere Particles (ISPs) was added to the emulsion PCR master mix and the emulsion was generated using an IKADT-20 mixer (Life Technologies). Next, ISPs were recovered and template-positive ISPs enriched for using Dynabeads MyOne Streptavidin C1 beads (Life Technologies). ISP enrichment was confirmed using the Qubit 2.0 fluorometer (Life Technologies). Sequencing was undertaken using 316 chips on the Ion Torrent PGM for 65 cycles and barcoding was used for these samples. The Ion Sequencing Kit v2.0 was used for sequencing reactions, following the recommended protocol (Part Number 4469714 Rev. B).

### Variant Calling

Data from the PGM runs were processed initially using the Ion Torrent platform-specific pipeline software Torrent Suite to generate sequence reads, trim adapter sequences, filter, and remove poor signal-profile reads. Initial variant calling from the Ion AmpliSeq sequencing data was generated using Torrent Suite Software v3.0 with a plug-in “variant caller v3.0” program. In order to eliminate erroneous base calling, several filtering steps were used to generate final variant calling (**Fig. S1**). The first filter was set at an average depth of total coverage of >100, an each variant coverage of >20, a variant frequency of each sample >5%, and P-value <0.01. The second filter was employed by visually examining mutations using Integrative Genomics Viewer (IGV) software (http//www.broadinstitute.org/igv) or Samtools software SAMtools software (http://samtools.sourceforge.net), as well as by filtering out possible strand-specific errors, ie. a mutation only detected in either “+” or “−” strand, but not in both strands of DNA. The third filtering step was set as variants within 727 hotspots, according to the manufacturer' instructions. The last filter step was to eliminate variants in amplicon AMPL339432 (PIK3CA, exon13, chr3:178938822-178938906), which is not uniquely matched in the human genome. From our sequencing runs using the Ion Ampliseq Cancer Panel, false deletion data were generated from the JAK2 gene locus and thus the sequencing data from this locus were excluded from further analysis.

### Somatic mutations

Detected mutations were compared to variants in the 1000 Genomes Project [12] and 6500 exomes of the National Heart, Lung, and Blood Institute Exome Sequencing Project [13] to distinguish somatic mutations and germline mutations.

### Bioinformatical and experimental validation

We used the COSMIC (version 64) [14], MyCancerGenome database (http://www.mycancergenome.org/) and some published literatures to assess reappearing mutations (**Table S1**). Additionally, some detected missense mutations were confirmed by Sanger's sequencing. (**Table S2**).

### Statistical analysis

We selected reappearing somatic missense/in-del mutations of gastric adenocarcinoma to perform the statistical analysis.

### Sequencing data

The dataset has been deposited to the NIH Sequence Read Archive, and the accession number is SRP040898.

## Results

### Ion Ampliseq sequencing of human gastric adenocarcinomas

**Figure 1:**
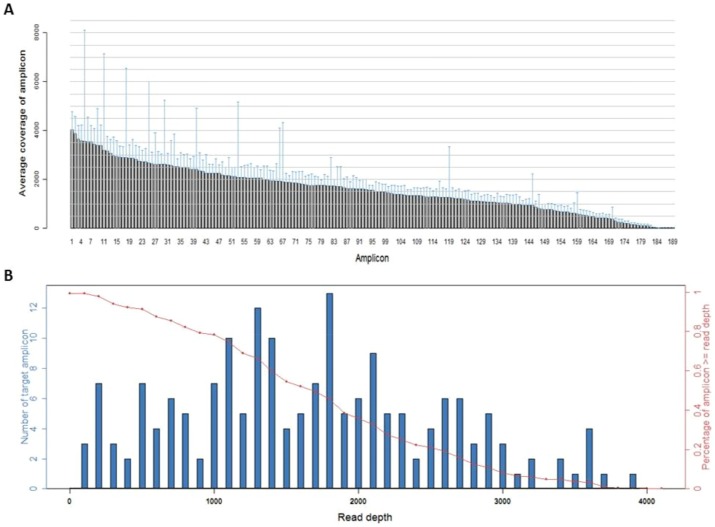
Sequence read distribution across 189 amplicons generated from 238 FFPE specimens, normalized to 300,000 reads per sample. A. Distribution of average coverage of each amplicon. Data are shown as mean ± SD. B. Number of amplicons with a given read depth, sorted in bins of 100 reads. Blue bars represent number of target amplicons within read depth, red line presents % of target amplicons ≥ read depth.

A total of 238 human gastric adenocarcinoma samples (**Table 1**) was analyzed using Ion Ampliseq Cancer Panel to identify mutation in 737 loci of 45 oncogenes and tumor suppressor genes in human gastric adenocarcinomas (**Fig. 1A**). Due to possible false base calling generated by the Ion Torrent sequencing technology, several sets of filters were used in order to yield reliable variant calling from the initial sequencing data, as described in the Materials and Methods. The mean read length was 75 bp. The average of sequence per sample was approximate 22 Mb. With normalization to 300,000 reads per specimen, there was an average of 1630 reads per amplicon (range, 21 to 4027) (**Fig. 1A**), 180/189 (95.2%) amplicons averaged at least 100 reads, and 170/189 (89.9%) amplicons averaged at least 300 reads (**Fig. 1B**).

**Table 1:** Mutations (including missense point mutations/deletion/insertion) frequencies in 45 genes (737 loci) in intestinal and diffuse GA samples (based on LAUREN classification).

Genes	Number of samples with mutations (Mutation frequency in 238 samples)	Number of 1 samples with mutations (Mutation frequency in 103 intestinal GA samples)	Number of 2 samples with mutations (Mutation frequency in 135 diffuse GA samples)
ABL1	0(0.0%)	0(0.0%)	0(0.0%)
AKT1	0(0.0%)	0(0.0%)	0(0.0%)
ALK	0(0.0%)	0(0.0%)	0(0.0%)
APC	1(0.4%)	1(1.0%)	0(0.0%)
ATM	0(0.0%)	0(0.0%)	0(0.0%)
BRAF	2(0.8%)	1(1.0%)	1(0.7%)
CDH1	0(0.0%)	0(0.0%)	0(0.0%)
CDKN2A	0(0.0%)	0(0.0%)	0(0.0%)
CSF1R	0(0.0%)	0(0.0%)	0(0.0%)
CTNNB1	0(0.0%)	0(0.0%)	0(0.0%)
EGFR	0(0.0%)	0(0.0%)	0(0.0%)
ERBB2	1(0.4%)	0(0.0%)	1(0.7%)
ERBB4	0(0.0%)	0(0.0%)	0(0.0%)
FBXW7	1(0.4%)	1(1.0%)	0(0.0%)
FGFR1	0(0.0%)	0(0.0%)	0(0.0%)
FGFR2	0(0.0%)	0(0.0%)	0(0.0%)
FGFR3	0(0.0%)	0(0.0%)	0(0.0%)
FLT3	0(0.0%)	0(0.0%)	0(0.0%)
GNAS	0(0.0%)	0(0.0%)	0(0.0%)
HNF1A	0(0.0%)	0(0.0%)	0(0.0%)
HRAS	0(0.0%)	0(0.0%)	0(0.0%)
IDH1	0(0.0%)	0(0.0%)	0(0.0%)
JAK3	0(0.0%)	0(0.0%)	0(0.0%)
KDR	0(0.0%)	0(0.0%)	0(0.0%)
KIT	2(0.8%)	1(1.0%)	1(0.7%)
KRAS	1(0.4%)	0(0.0%)	1(0.7%)
MET	0(0.0%)	0(0.0%)	0(0.0%)
MLH1	0(0.0%)	0(0.0%)	0(0.0%)
MPL	0(0.0%)	0(0.0%)	0(0.0%)
NOTCH1	0(0.0%)	0(0.0%)	0(0.0%)
NPM1	0(0.0%)	0(0.0%)	0(0.0%)
NRAS	0(0.0%)	0(0.0%)	0(0.0%)
PDGFRA	1(0.4%)	1(1.0%)	0(0.0%)
PIK3CA	2(0.8%)	0(0.0%)	2(1.5%)
PTEN	1(0.4%)	0(0.0%)	1(0.7%)
PTPN11	0(0.0%)	0(0.0%)	0(0.0%)
RB1	1(0.4%)	0(0.0%)	1(0.7%)
RET	0(0.0%)	0(0.0%)	0(0.0%)
SMAD4	2(0.8%)	1(1.0%)	1(0.7%)
SMARCB1	0(0.0%)	0(0.0%)	0(0.0%)
SMO	0(0.0%)	0(0.0%)	0(0.0%)
SRC	0(0.0%)	0(0.0%)	0(0.0%)
STK11	0(0.0%)	0(0.0%)	0(0.0%)
TP53	23(9.7%)	13(12.6%)	10(7.4%)
VHL	0(0.0%)	0(0.0%)	0(0.0%)

In this study, we sequenced 238 human gastric adenocarcinoma samples with an average coverage depth of the targeted loci >100 (**Fig. 1A**). Tumors in our sample set was classified into diffuse or intestinal type based on Lauren classification (**Table 1**). Using a strict standard variant calling, we identified mutations in the following genes (**Table 2**): APC, BRAF, ERBB2 FBXW7, KIT, PDGFRA, PIK3CA, PTEN, RB1, SMAD4, and a high incurrence of mutation along the TP53 gene. Detailed frequencies of missense, point mutations, insertions, and deletions profiled on the 737 loci of 45 tumor suppressor genes and oncogenes of 238 gastric adenocarcinoma samples is provided in the **Table S1**. Our sample set included tumors scored at different stages of disease (Ia, Ib, II, IIIa, IIIb, IV) based on the AJCC/TNM cancer staging system (**Table 3**), and were also at different differentiation potentials (**Table 4**). Detailed sequencing analysis in the exons and functional domains of TP53 is outlined below.

**Table 2:** Mutations (including missense point mutations/deletion/insertion) frequencies in 45 genes (737 loci) in female and male GAs.

Genes	Number of samples with mutations (Mutation frequency in 238 samples)	Number of female samples with mutations (Mutation frequency in 51 female samples)	Number of male samples with mutations (Mutation frequency in 187 male samples)
ABL1	0(0.0%)	0(0.0%)	0(0.0%)
AKT1	0(0.0%)	0(0.0%)	0(0.0%)
ALK	0(0.0%)	0(0.0%)	0(0.0%)
APC	1(0.4%)	0(0.0%)	1(0.5%)
ATM	0(0.0%)	0(0.0%)	0(0.0%)
BRAF	2(0.8%)	0(0.0%)	2(1.1%)
CDH1	0(0.0%)	0(0.0%)	0(0.0%)
CDKN2A	0(0.0%)	0(0.0%)	0(0.0%)
CSF1R	0(0.0%)	0(0.0%)	0(0.0%)
CTNNB1	0(0.0%)	0(0.0%)	0(0.0%)
EGFR	0(0.0%)	0(0.0%)	0(0.0%)
ERBB2	1(0.4%)	1(2.0%)	0(0.0%)
ERBB4	0(0.0%)	0(0.0%)	0(0.0%)
FBXW7	1(0.4%)	0(0.0%)	1(0.5%)
FGFR1	0(0.0%)	0(0.0%)	0(0.0%)
FGFR2	0(0.0%)	0(0.0%)	0(0.0%)
FGFR3	0(0.0%)	0(0.0%)	0(0.0%)
FLT3	0(0.0%)	0(0.0%)	0(0.0%)
GNAS	0(0.0%)	0(0.0%)	0(0.0%)
HNF1A	0(0.0%)	0(0.0%)	0(0.0%)
HRAS	0(0.0%)	0(0.0%)	0(0.0%)
IDH1	0(0.0%)	0(0.0%)	0(0.0%)
JAK3	0(0.0%)	0(0.0%)	0(0.0%)
KDR	0(0.0%)	0(0.0%)	0(0.0%)
KIT	2(0.8%)	0(0.0%)	2(1.1%)
KRAS	1(0.4%)	0(0.0%)	1(0.5%)
MET	0(0.0%)	0(0.0%)	0(0.0%)
MLH1	0(0.0%)	0(0.0%)	0(0.0%)
MPL	0(0.0%)	0(0.0%)	0(0.0%)
NOTCH1	0(0.0%)	0(0.0%)	0(0.0%)
NPM1	0(0.0%)	0(0.0%)	0(0.0%)
NRAS	0(0.0%)	0(0.0%)	0(0.0%)
PDGFRA	1(0.4%)	0(0.0%)	1(0.5%)
PIK3CA	2(0.8%)	1(2.0%)	1(0.5%)
PTEN	1(0.4%)	1(2.0%)	0(0.0%)
PTPN11	0(0.0%)	0(0.0%)	0(0.0%)
RB1	1(0.4%)	1(2.0%)	0(0.0%)
RET	0(0.0%)	0(0.0%)	0(0.0%)
SMAD4	2(0.8%)	0(0.0%)	2(1.1%)
SMARCB1	0(0.0%)	0(0.0%)	0(0.0%)
SMO	0(0.0%)	0(0.0%)	0(0.0%)
SRC	0(0.0%)	0(0.0%)	0(0.0%)
STK11	0(0.0%)	0(0.0%)	0(0.0%)
TP53	23(9.7%)	7(13.7%)	16(8.6%)
VHL	0(0.0%)	0(0.0%)	0(0.0%)

**Table 3:** Mutations (including missense point mutations/deletion/insertion) frequencies in 45 genes (737 loci) at different AJCC staging.

Genes	Number of samples with mutations (Mutation frequency in 238 samples)	Number of stage 1A samples with mutations (Mutation frequency in 26 samples)	Number of stage 1B samples with mutations (Mutation frequency in 34 samples)	Number of stage 2 samples with mutations (Mutation frequency in 49 samples)	Number of stage 3A samples with mutations (Mutation frequency in 79 samples)	Number of stage 3B samples with mutations (Mutation frequency in 29 samples)	Number of stage 4 samples with mutations (Mutation frequency in 21 samples)
ABL1	0(0.0%)	0(0.0%)	0(0.0%)	0(0.0%)	0(0.0%)	0(0.0%)	0(0.0%)
AKT1	0(0.0%)	0(0.0%)	0(0.0%)	0(0.0%)	0(0.0%)	0(0.0%)	0(0.0%)
ALK	0(0.0%)	0(0.0%)	0(0.0%)	0(0.0%)	0(0.0%)	0(0.0%)	0(0.0%)
APC	1(0.4%)	0(0.0%)	0(0.0%)	0(0.0%)	1(1.3%)	0(0.0%)	0(0.0%)
ATM	0(0.0%)	0(0.0%)	0(0.0%)	0(0.0%)	0(0.0%)	0(0.0%)	0(0.0%)
BRAF	2(0.8%)	0(0.0%)	0(0.0%)	1(2.0%)	0(0.0%)	1(3.4%)	0(0.0%)
CDH1	0(0.0%)	0(0.0%)	0(0.0%)	0(0.0%)	0(0.0%)	0(0.0%)	0(0.0%)
CDKN2A	0(0.0%)	0(0.0%)	0(0.0%)	0(0.0%)	0(0.0%)	0(0.0%)	0(0.0%)
CSF1R	0(0.0%)	0(0.0%)	0(0.0%)	0(0.0%)	0(0.0%)	0(0.0%)	0(0.0%)
CTNNB1	0(0.0%)	0(0.0%)	0(0.0%)	0(0.0%)	0(0.0%)	0(0.0%)	0(0.0%)
EGFR	0(0.0%)	0(0.0%)	0(0.0%)	0(0.0%)	0(0.0%)	0(0.0%)	0(0.0%)
ERBB2	1(0.4%)	0(0.0%)	0(0.0%)	0(0.0%)	1(1.3%)	0(0.0%)	0(0.0%)
ERBB4	0(0.0%)	0(0.0%)	0(0.0%)	0(0.0%)	0(0.0%)	0(0.0%)	0(0.0%)
FBXW7	1(0.4%)	0(0.0%)	0(0.0%)	0(0.0%)	1(1.3%)	0(0.0%)	0(0.0%)
FGFR1	0(0.0%)	0(0.0%)	0(0.0%)	0(0.0%)	0(0.0%)	0(0.0%)	0(0.0%)
FGFR2	0(0.0%)	0(0.0%)	0(0.0%)	0(0.0%)	0(0.0%)	0(0.0%)	0(0.0%)
FGFR3	0(0.0%)	0(0.0%)	0(0.0%)	0(0.0%)	0(0.0%)	0(0.0%)	0(0.0%)
FLT3	0(0.0%)	0(0.0%)	0(0.0%)	0(0.0%)	0(0.0%)	0(0.0%)	0(0.0%)
GNAS	0(0.0%)	0(0.0%)	0(0.0%)	0(0.0%)	0(0.0%)	0(0.0%)	0(0.0%)
HNF1A	0(0.0%)	0(0.0%)	0(0.0%)	0(0.0%)	0(0.0%)	0(0.0%)	0(0.0%)
HRAS	0(0.0%)	0(0.0%)	0(0.0%)	0(0.0%)	0(0.0%)	0(0.0%)	0(0.0%)
IDH1	0(0.0%)	0(0.0%)	0(0.0%)	0(0.0%)	0(0.0%)	0(0.0%)	0(0.0%)
JAK3	0(0.0%)	0(0.0%)	0(0.0%)	0(0.0%)	0(0.0%)	0(0.0%)	0(0.0%)
KDR	0(0.0%)	0(0.0%)	0(0.0%)	0(0.0%)	0(0.0%)	0(0.0%)	0(0.0%)
KIT	2(0.8%)	0(0.0%)	0(0.0%)	1(2.0%)	1(1.3%)	0(0.0%)	0(0.0%)
KRAS	1(0.4%)	0(0.0%)	0(0.0%)	0(0.0%)	1(1.3%)	0(0.0%)	0(0.0%)
MET	0(0.0%)	0(0.0%)	0(0.0%)	0(0.0%)	0(0.0%)	0(0.0%)	0(0.0%)
MLH1	0(0.0%)	0(0.0%)	0(0.0%)	0(0.0%)	0(0.0%)	0(0.0%)	0(0.0%)
MPL	0(0.0%)	0(0.0%)	0(0.0%)	0(0.0%)	0(0.0%)	0(0.0%)	0(0.0%)
NOTCH1	0(0.0%)	0(0.0%)	0(0.0%)	0(0.0%)	0(0.0%)	0(0.0%)	0(0.0%)
NPM1	0(0.0%)	0(0.0%)	0(0.0%)	0(0.0%)	0(0.0%)	0(0.0%)	0(0.0%)
NRAS	0(0.0%)	0(0.0%)	0(0.0%)	0(0.0%)	0(0.0%)	0(0.0%)	0(0.0%)
PDGFRA	1(0.4%)	0(0.0%)	0(0.0%)	1(2.0%)	0(0.0%)	0(0.0%)	0(0.0%)
PIK3CA	2(0.8%)	0(0.0%)	1(2.9%)	0(0.0%)	1(1.3%)	0(0.0%)	0(0.0%)
PTEN	1(0.4%)	0(0.0%)	0(0.0%)	0(0.0%)	1(1.3%)	0(0.0%)	0(0.0%)
PTPN11	0(0.0%)	0(0.0%)	0(0.0%)	0(0.0%)	0(0.0%)	0(0.0%)	0(0.0%)
RB1	1(0.4%)	0(0.0%)	0(0.0%)	0(0.0%)	0(0.0%)	0(0.0%)	1(4.8%)
RET	0(0.0%)	0(0.0%)	0(0.0%)	0(0.0%)	0(0.0%)	0(0.0%)	0(0.0%)
SMAD4	2(0.8%)	0(0.0%)	0(0.0%)	1(2.0%)	1(1.3%)	0(0.0%)	0(0.0%)
SMARCB1	0(0.0%)	0(0.0%)	0(0.0%)	0(0.0%)	0(0.0%)	0(0.0%)	0(0.0%)
SMO	0(0.0%)	0(0.0%)	0(0.0%)	0(0.0%)	0(0.0%)	0(0.0%)	0(0.0%)
SRC	0(0.0%)	0(0.0%)	0(0.0%)	0(0.0%)	0(0.0%)	0(0.0%)	0(0.0%)
STK11	0(0.0%)	0(0.0%)	0(0.0%)	0(0.0%)	0(0.0%)	0(0.0%)	0(0.0%)
TP53	23(9.7%)	3(11.5%)	3(8.8%)	7(14.3%)	8(10.1%)	0(0.0%)	2(9.5%)
VHL	0(0.0%)	0(0.0%)	0(0.0%)	0(0.0%)	0(0.0%)	0(0.0%)	0(0.0%)

**Table 4:** Mutations (including missense point mutations/deletion/insertion) frequencies in 45 genes (737 loci) of different differentiation types of GAs.

Genes	Number of samples with mutations in 238 samples (Mutation frequency)	Number of low differentiation samples with mutations (Mutation frequency in 125 samples)	Number of middle-low differentiation samples with mutations (Mutation frequency in 14 samples)	Number of middle differentiation samples with mutations (Mutation frequency in 84 samples)	Number of unknown samples with mutations (Mutation frequency in 15 unknown samples)
ABL1	0(0.0%)	0(0.0%)	0(0.0%)	0(0.0%)	0(0.0%)
AKT1	0(0.0%)	0(0.0%)	0(0.0%)	0(0.0%)	0(0.0%)
ALK	0(0.0%)	0(0.0%)	0(0.0%)	0(0.0%)	0(0.0%)
APC	1(0.4%)	1(0.8%)	0(0.0%)	0(0.0%)	0(0.0%)
ATM	0(0.0%)	0(0.0%)	0(0.0%)	0(0.0%)	0(0.0%)
BRAF	2(0.8%)	1(0.8%)	0(0.0%)	1(1.2%)	0(0.0%)
CDH1	0(0.0%)	0(0.0%)	0(0.0%)	0(0.0%)	0(0.0%)
CDKN2A	0(0.0%)	0(0.0%)	0(0.0%)	0(0.0%)	0(0.0%)
CSF1R	0(0.0%)	0(0.0%)	0(0.0%)	0(0.0%)	0(0.0%)
CTNNB1	0(0.0%)	0(0.0%)	0(0.0%)	0(0.0%)	0(0.0%)
EGFR	0(0.0%)	0(0.0%)	0(0.0%)	0(0.0%)	0(0.0%)
ERBB2	1(0.4%)	1(0.8%)	0(0.0%)	0(0.0%)	0(0.0%)
ERBB4	0(0.0%)	0(0.0%)	0(0.0%)	0(0.0%)	0(0.0%)
FBXW7	1(0.4%)	1(0.8%)	0(0.0%)	0(0.0%)	0(0.0%)
FGFR1	0(0.0%)	0(0.0%)	0(0.0%)	0(0.0%)	0(0.0%)
FGFR2	0(0.0%)	0(0.0%)	0(0.0%)	0(0.0%)	0(0.0%)
FGFR3	0(0.0%)	0(0.0%)	0(0.0%)	0(0.0%)	0(0.0%)
FLT3	0(0.0%)	0(0.0%)	0(0.0%)	0(0.0%)	0(0.0%)
GNAS	0(0.0%)	0(0.0%)	0(0.0%)	0(0.0%)	0(0.0%)
HNF1A	0(0.0%)	0(0.0%)	0(0.0%)	0(0.0%)	0(0.0%)
HRAS	0(0.0%)	0(0.0%)	0(0.0%)	0(0.0%)	0(0.0%)
IDH1	0(0.0%)	0(0.0%)	0(0.0%)	0(0.0%)	0(0.0%)
JAK3	0(0.0%)	0(0.0%)	0(0.0%)	0(0.0%)	0(0.0%)
KDR	0(0.0%)	0(0.0%)	0(0.0%)	0(0.0%)	0(0.0%)
KIT	2(0.8%)	2(1.6%)	0(0.0%)	0(0.0%)	0(0.0%)
KRAS	1(0.4%)	1(0.8%)	0(0.0%)	0(0.0%)	0(0.0%)
MET	0(0.0%)	0(0.0%)	0(0.0%)	0(0.0%)	0(0.0%)
MLH1	0(0.0%)	0(0.0%)	0(0.0%)	0(0.0%)	0(0.0%)
MPL	0(0.0%)	0(0.0%)	0(0.0%)	0(0.0%)	0(0.0%)
NOTCH1	0(0.0%)	0(0.0%)	0(0.0%)	0(0.0%)	0(0.0%)
NPM1	0(0.0%)	0(0.0%)	0(0.0%)	0(0.0%)	0(0.0%)
NRAS	0(0.0%)	0(0.0%)	0(0.0%)	0(0.0%)	0(0.0%)
PDGFRA	1(0.4%)	0(0.0%)	0(0.0%)	1(1.2%)	0(0.0%)
PIK3CA	2(0.8%)	0(0.0%)	1(7.1%)	0(0.0%)	1(6.7%)
PTEN	1(0.4%)	1(0.8%)	0(0.0%)	0(0.0%)	0(0.0%)
PTPN11	0(0.0%)	0(0.0%)	0(0.0%)	0(0.0%)	0(0.0%)
RB1	1(0.4%)	1(0.8%)	0(0.0%)	0(0.0%)	0(0.0%)
RET	0(0.0%)	0(0.0%)	0(0.0%)	0(0.0%)	0(0.0%)
SMAD4	2(0.8%)	1(0.8%)	0(0.0%)	1(1.2%)	0(0.0%)
SMARCB1	0(0.0%)	0(0.0%)	0(0.0%)	0(0.0%)	0(0.0%)
SMO	0(0.0%)	0(0.0%)	0(0.0%)	0(0.0%)	0(0.0%)
SRC	0(0.0%)	0(0.0%)	0(0.0%)	0(0.0%)	0(0.0%)
STK11	0(0.0%)	0(0.0%)	0(0.0%)	0(0.0%)	0(0.0%)
TP53	23(9.7%)	10(8.0%)	0(0.0%)	10(11.9%)	3(20.0%)
VHL	0(0.0%)	0(0.0%)	0(0.0%)	0(0.0%)	0(0.0%)

### Missense mutation distribution in the exons and functional domains of TP53

**Figure 2:**
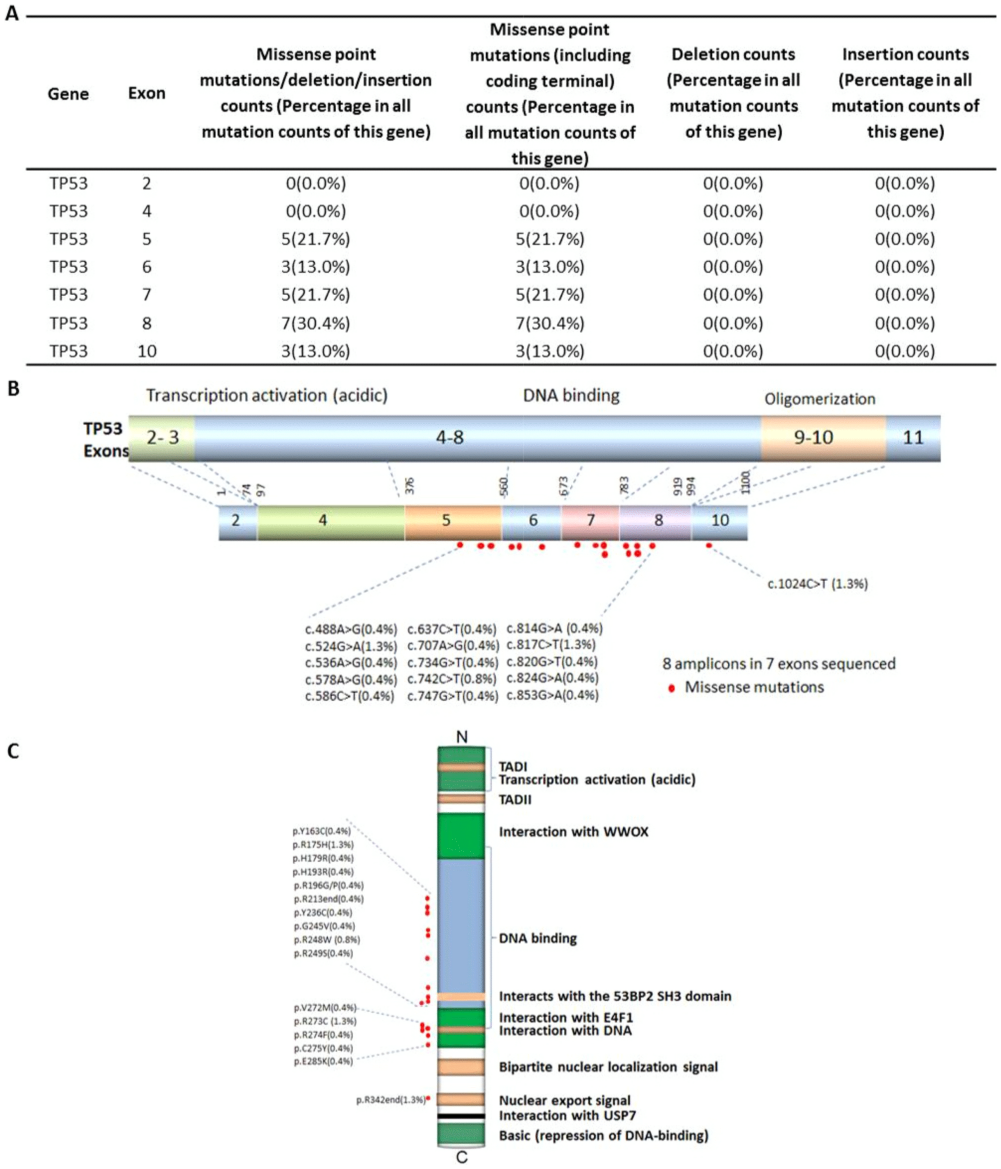
Missense mutation distribution in the exons and function domains of TP53. A. Frequencies of detected mutations in different exons. B. Mutation distribution in exons. C. Mutation distribution in functional domains.

Abnormality of the TP53 gene is one of the most common events in gastric cancers and plays an important role in the tumorigenesis of gastric epithelial cells. The p53 tumor suppressor gene is located on 17p13 chromosome and spans 20 kb genomic DNA, encompassing 11 exons that encode for a 53 kDa phosphoprotein [15]. 12.6% of TP53-associated gastric cancers were intestinal and 7.4% were diffuse (**Table 1**). 11.9% of TP53 mutations were associated with ‘mid differentiation’ cancers and 8.0% of TP53 mutations were ‘low differentiation’ cancers (**Table 4**). In our sample set, 7.4% of TP53-associated gastric cancers were at stage 3 and 9.5% were at stage 4 according to the AJCC cancer staging system (**Table 3**). Most TP53 mutations cluster in the TP53 DNA-binding domain, which encompasses exons 5 through 8 and spans approximately 180 codons or 540 nucleotides and is not limited to a few particular sequences or codons [16]. In our sample set, the mutations incurred along the DNA-binding domain encoded from exons 5 (21.7%), 6 (13.0%), 7 (21.7%), and 8 (30.4%), and along the oligomerization domain encoded from exon 10 (13.0%), and all were missense point mutations (**Figs. 2A–C**). Most TP53 missense mutations led to the synthesis of a stable protein, which lacks its specific DNA-binding and transactivation function and accumulates in the nucleus of cells. Such mutant proteins become inactive and lack the ability to transactivate the downstream target genes that regulate cell cycle and apoptosis [17]. Apart from these mutations affecting the role of TP53 as a tumor-suppressor protein, TP53 mutations also endow the mutant protein with ‘gain-of-function’ (GOF) activities, which can contribute actively to various stages of tumor progression, including distant metastases, and to increased resistance to anticancer treatments [15], [18], [19].

### Multiple mutations and mutation hot spots in gastric adenocarcinomas

Clinical success with individualized combination therapy relies on the identification of mutational combinations and patterns for co-administration of a single or combination of target agents against the detected mutational combinations. Some of the mutations detected in our tumor group through sequencing analysis were not only recurrent and frequent but also occurred in combination with other mutations. 13.6% of samples had at least one or more missense mutations, 1.70% had at least two or more missense mutations, 0.4% had at least three or more missense mutations and 86.1% of samples incurred no deleterious mutations in any of the screened 737 loci of the potential tumor suppressor genes and oncogenes (**Table 5**).

**Table 5:** Single and multiple mutations in 238 GAs.

Mutations combination (including missense point mutations/deletion/insertion)	Number of samples with mutation combinations	Percentage in all sequenced samples
Single and more	33	13.60%
Double and more	4	1.70%
Three and more	1	0.40%
Four and more	0	0.00%
Five and more	0	0.00%
No. missense, deletion, insert or substitution-nonsense	205	86.10%

## Discussion

Gastric adenocarcinoma, the most common type of gastric cancer, is heterogeneous and its incidence and cause varies widely with geographical regions, gender, ethnicity, and diet [20]. The infectious agent *Helicobacter pylori* is associated with chronic atrophic gastritis, an inflammatory precursor of gastric adenocarcinoma [20]. While *H. pylori* colonizes the gastric tract of most of the world's population and induces mutations and genomic instability in host DNA, only individuals with a complex risk profile tend to develop cancer [21].

In this study we have used Ion Ampliseq Cancer Panel to sequence 737 loci in 45 cancer-related genes, mainly oncogenes and tumor suppressor genes, of 238 human gastric adenocarcinoma samples. 23 out of 238 samples incurred mutations along the TP53 gene. Other genes such as BRAF, APC, FBXW7, ERBB2, KRAS, PIK3CA, PTEN, RB1, and SMAD4 incurred mutations in only 1–2 out of 238 samples. The incurrence of TP53 mutation and that of other genes such as KRAS less frequently mutated in our sample set was consistent to that of previous reports on mutation screening for gastric cancers [22]–[24]. Wang et al. reported that certain subtypes of gastric cancers accumulated mutations in ARID1A gene, but is negatively associated with mutations in TP53 [22]. Another study by Nagarajan et al. reported PAPPA as a recurrently mutated gene in TP53 wild-type gastric cancer [24]. It is reported that mutations in TP53 is not common in ovarian, endometrial, kidney, or pancreatic cancers, but these frequently accumulate mutations in chromatin-modifying genes, suggesting the existence of alternative pathways of carcinogenesis between these subsets of cancer [25]–[28]. Although ARID1A is not included in our sequencing panel, it is possible that gastric adenocarcinomas with TP53 mutations in our sample set follow an alternative pathway to that chromatin modifying gene-associated cancers. Thus, this study indicates the necessity of sequencing individual human adenocarcinomas in order to match the use of a single targeted drug or the combinational use of two or more targeted drugs against the individual adenocarcinoma-specific mutations.

The genomic landscape of gastric cancers is recently being extensively analyzed, and while common mutations, amplifications, and deletions have been profiled already, detection of novel mutations and their co-occurring patterns have gained importance in the recent times [29]. For example, BRAF, KRAS, and PIK3CA, a set of rarely identified mutations in sporadic gastric cancers, have recently been reported in the microsatellite subsets of cancer [30]. HER2 (ERBB2) overexpression varied according to gastric cancer subtypes and targeting HER2 through the use of a humanized monoclonal antibody Trastuzumab (Herceptin) has been very successful in the treatment of HER2-overexpressed gastric cancers [31]. Overexpression of EGFR is associated with gastric cancer and there are several drugs such as Gefitinib, Erlotinib, and Ceutuximab that target EGFR mutations either in combination or as single agents [32]–[35]. High VEGF levels in the serum is associated with the prognosis of advanced gastric cancer; bevacizumab, an anti-VEGF monoclonal antibody, helps enhance survival rate of these patients [36], [37]. PI3K/mTOR pathway activation has been demonstrated in gastric cancer [38] and everolimus (Afinitor) has shown some significant response in the gastric cancer patients through targeting the mutations associated with PI3K/mTOR pathway [39], [40]. Overexpression and amplification of MET occurs in many gastric cancers and one study has shown PHA-665752 as a potential target for MET amplification [41] and Onartuzumab, a humanized anti-MET antibody, has been shown to produce sustained responses against MET copy number mutations [42]. Recent sequencing studies have shown that up to 35% of gastric cancers have p53 mutations and they are known for their resistance to chemotherapeutic drugs such as Camptothecin analogues due to the activation of cell cycle checkpoints which induces permanent cell cycle arrest at the G2 phase instead of causing cell death [43]. To circumvent these issues, chk1 inhibitors are currently gaining attraction, with UCN-01 as one such drug under clinical development [44].

Since there are multiple mutations in individual tumors, and each tumor has a unique set of mutations, identification and characterization of the molecular alterations underlying individual gastric adenocarcinomas is a critical step for developing effective and personalized therapies. Until recently, identifying genetic mutations on an individual basis by DNA sequencing remained impractical. The recent advance of new next-generation DNA sequencing technologies, such as the semiconductor-based Ion Torrent sequencing platform, makes DNA sequencing cheaper, faster, and more reliable. Our study shows distinctive patterns and combinations of mutations in the gastric adenocarcinoma genome of these Chinese patients. Genomic profiling and identification of specific mutation patterns and designing personalized drug targets and treatment regimens against those cancer mutations can be very useful for personalized therapy. There are many novel compounds available today targeting different molecular pathways of gastric cancer such as HER2, EGFR, MET, FGFR, and PI3K/MTOR, which could potentially be used for the treatment of gastric adenocarcinomas. Hence, we believe that a faster and more cost-effective, accurate high-throughput genomic profiling tool such as Ion Torrent sequencing employed in our current studies will facilitate the implementation of tailored and personalized therapies in the near future.

## Supporting Information

Figure S1 **Filter process of variants.** (a) Strand-biased variants were eliminated using Integrative Genomics Viewer (IGV) software (http//www.broadinstitute.org/igv); (b) Variants in AMPL339432 should be eliminated, because this amplicon is not unique matched to PIK3CA in human genome; (c) All of our statistical analysis was based on the data in blue box.
(DOCX)

Table S1 **Frequencies of point mutations, insertion, and deletion mutations in 737 loci of 45 genes in 238 GAs.**
(DOCX)

Table S2 **Confirmation of missense mutations by Sanger sequencing.**
(DOCX)
