# Supplementary figures and images for: Genetic Mutation Analysis of Human Gastric Adenocarcinomas Using Ion Torrent Sequencing Platform
Source: PLoS One. 2014 Jul 15;9(7):e100442. doi: 10.1371/journal.pone.0100442 (PMC4098916; doi:10.1371/journal.pone.0100442)

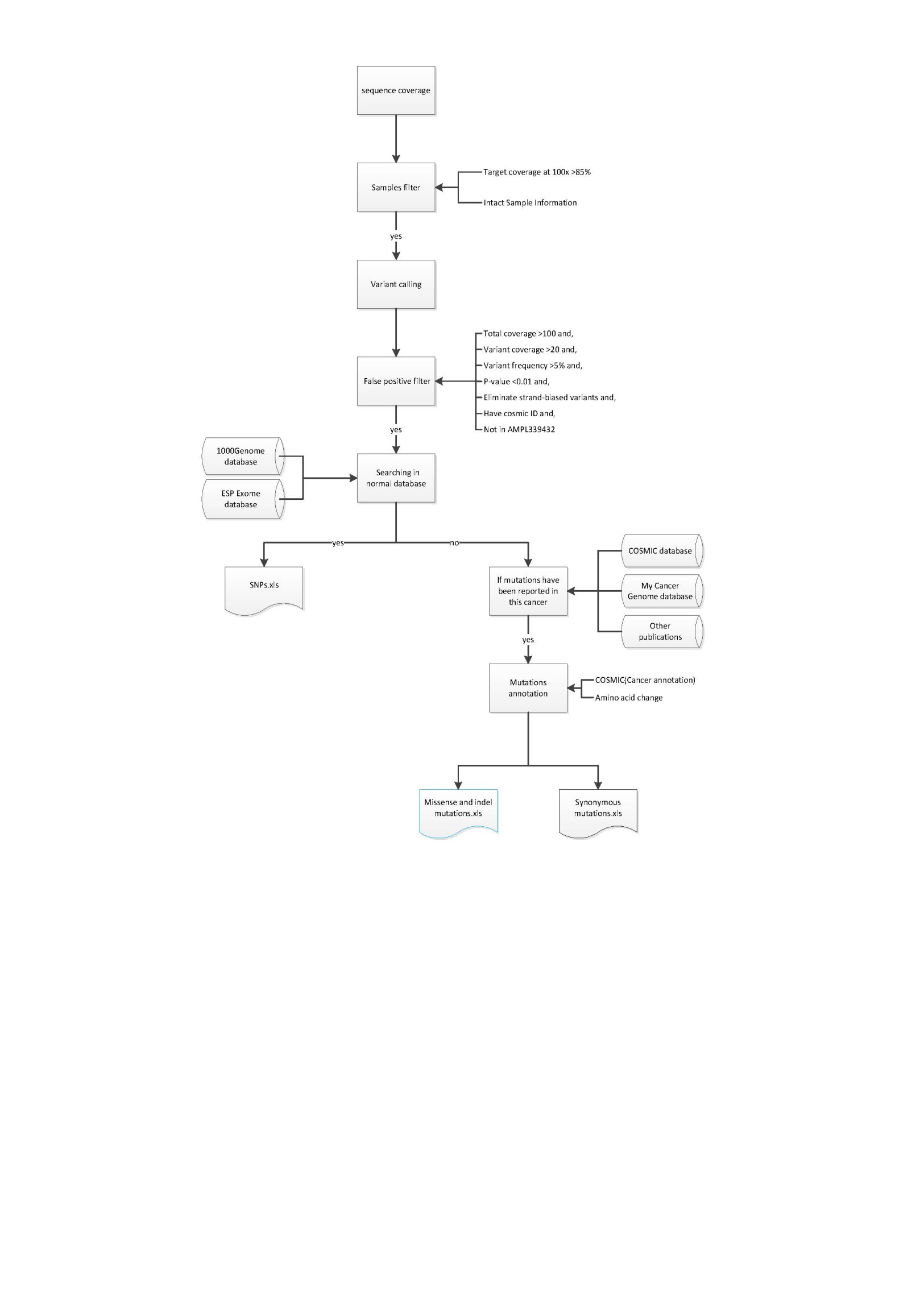
**Fig. S1: Filter process variants**

Supplement: Figure S1 — Filter process of variants. (a) Strand-biased variants were eliminated using Integrative Genomics Viewer (IGV) software (http//www.broadinstitute.org/igv); (b) Variants in AMPL339432 should be eliminated, because this amplicon is not unique matched to PIK3CA in human genome; (c) All of our statistical analysis was based on the data in blue box. (DOCX) [file pone.0100442.s001.docx]
