# Supplementary material for: Genetic Mutation Analysis of Human Gastric Adenocarcinomas Using Ion Torrent Sequencing Platform
Source: PLoS One. 2014 Jul 15;9(7):e100442. doi: 10.1371/journal.pone.0100442 (PMC4098916; doi:10.1371/journal.pone.0100442)
Supplement: Table S1 — Frequencies of point mutations, insertion, and deletion mutations in 737 loci of 45 genes in 238 GAs. (DOCX) [file pone.0100442.s002.docx]

**Table S1. Frequencies of point mutations, insertion and deletion mutations in 737 loci of 45 genes in 238 gastric adenocarcinoma patients.**

| **Gene Mutations** | **Number of samples with this mutation site** | **Number of samples with this mutation gene** | **Mutation Frequency** | **Gene mutation frequency in publications** | **Site mutation frequency in gene in publications** | **If reported in gastric adenocarcinoma in COSMIC database** | **If reported in gastric adenocarcinoma in MyCancerGenome** |
| --- | --- | --- | --- | --- | --- | --- | --- |
| APC c.2626C>T | 1 | 1 | 0.4% | 4% ^[^[^4^](#_ENREF_4)^]^(Korea) | - | NO | NO |
| BRAF c.1330C>T | 1 | 2 | 0.8% | 2.2%^[^[^5^](#_ENREF_5)^]^( Asia, Korea) | - | NO | NO |
| BRAF c.1796C>T | 1 |  |  |  | - | NO | NO |
| ERBB2 c.2329G>T | 1 | 1 | 0.4% | 5.0%^[^[^6^](#_ENREF_6)^]^（Korea） | 1.1%^[^[^6^](#_ENREF_6)^]^（Korea） | YES | NO |
| FBXW7 c.1394G>A | 1 | 1 | 0.4% | ～6%^[^[^7^](#_ENREF_7)^]^（Austria） |  | NO | NO |
| KIT c.1676T>A | 1 | 2 | 0.8% | - | - | NO | NO |
| KIT c.1676T>G | 1 |  |  |  | - | NO | NO |
| KRAS c.35G>A | 1 | 1 | 0.8% | - | - | YES | NO |
| PDGFRA c.2021C>T | 1 | 1 | 0.4% | - | - | NO | NO |
| PIK3CA c.1624G>A | 1 | 2 | 0.8% | 4.3%^[^[^8^](#_ENREF_8)^]^（HONGKONG） | 1.1%^[^[^8^](#_ENREF_8)^]^ | YES | NO |
| PIK3CA c.1633G>A | 1 |  |  |  |  | YES | NO |
| PTEN c.1003C>T | 1 | 1 | 0.4% | 10% in advanced gastric cancers^[^[^9^](#_ENREF_9)^]^（China） | - | YES | NO |
| RB1 c.1654C>T | 1 | 1 | 0.4% | - | methylation ^[^[^10^](#_ENREF_10)^]^ | NO | NO |
| SMAD4 c.1082G>A | 1 | 2 | 0.8% | ~2.9%^[^[^11^](#_ENREF_11)^]^（American） | - | NO | NO |
| SMAD4 c.733C>T | 1 |  |  |  | - | NO | NO |
| TP53 c.1024C>T | 3 | 23 | 9.7% | 37%～42%^[^[^12^](#_ENREF_12)^]^（Japan） | - | YES | NO |
| TP53 c.488A>G | 1 |  |  |  | - | YES | NO |
| TP53 c.524G>A | 3 |  |  |  | - | YES | NO |
| TP53 c.536A>G | 1 |  |  |  | - | NO | NO |
| TP53 c.578A>G | 1 |  |  |  | - | YES | NO |
| TP53 c.586C>T | 1 |  |  |  | - | YES | NO |
| TP53 c.637C>T | 1 |  |  |  | - | YES | NO |
| TP53 c.707A>G | 1 |  |  |  | - | YES | NO |
| TP53 c.734G>T | 1 |  |  |  | - | YES | NO |
| TP53 c.742C>T | 2 |  |  |  | - | YES | NO |
| TP53 c.747G>T | 1 |  |  |  | - | YES | NO |
| TP53 c.814G>A | 1 |  |  |  | - | YES | NO |
| TP53 c.817C>T | 3 |  |  |  | - | YES | NO |
| TP53 c.820G>T | 1 |  |  |  | - | NO | NO |
| TP53 c.824G>A | 1 |  |  |  | - | YES | NO |
| TP53 c.853G>A | 1 |  |  |  | - | YES | NO |
