# Supplementary material for: Genetic Mutation Analysis of Human Gastric Adenocarcinomas Using Ion Torrent Sequencing Platform
Source: PLoS One. 2014 Jul 15;9(7):e100442. doi: 10.1371/journal.pone.0100442 (PMC4098916; doi:10.1371/journal.pone.0100442)
Supplement: Table S2 — Confirmation of missense mutations by Sanger sequencing. (DOCX) [file pone.0100442.s003.docx]

**Table S2. Sanger results**

| **#** | **Sample ID** | **Cosmic ID** | **Gene** | **Mutation CDS** | **Mutation AA** | **Variant Frequency (%)** | **Sanger result Consistent** |
| --- | --- | --- | --- | --- | --- | --- | --- |
| 1 | 1D5 | COSM11063 | TP53 | c.711G>A | p.M237I | 17.68 | YES |
| 2 | 2B11 | COSM11073 | TP53 | c.1024C>T | p.R342// | 20.36 | Not sure |
| 3 | 1F1 | COSM10648 | TP53 | c.524G>A | p.R175H | 5.92 | YES |

Sanger figures

#1


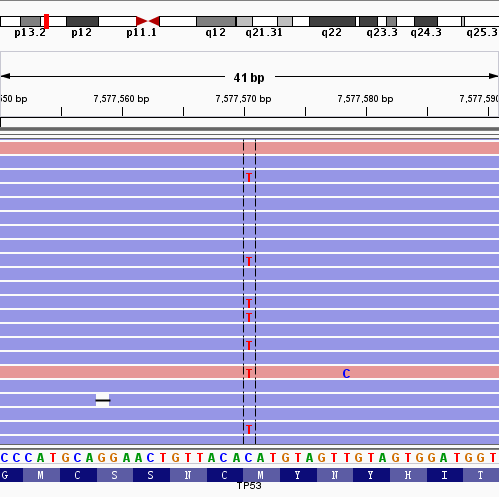


sense


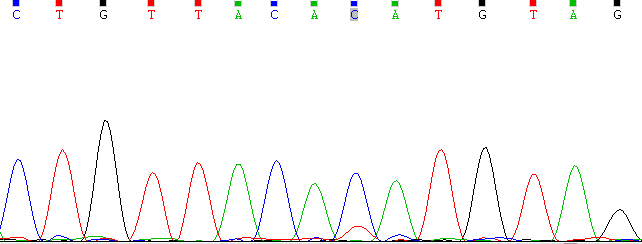


Sanger

Ion Torrent

#2


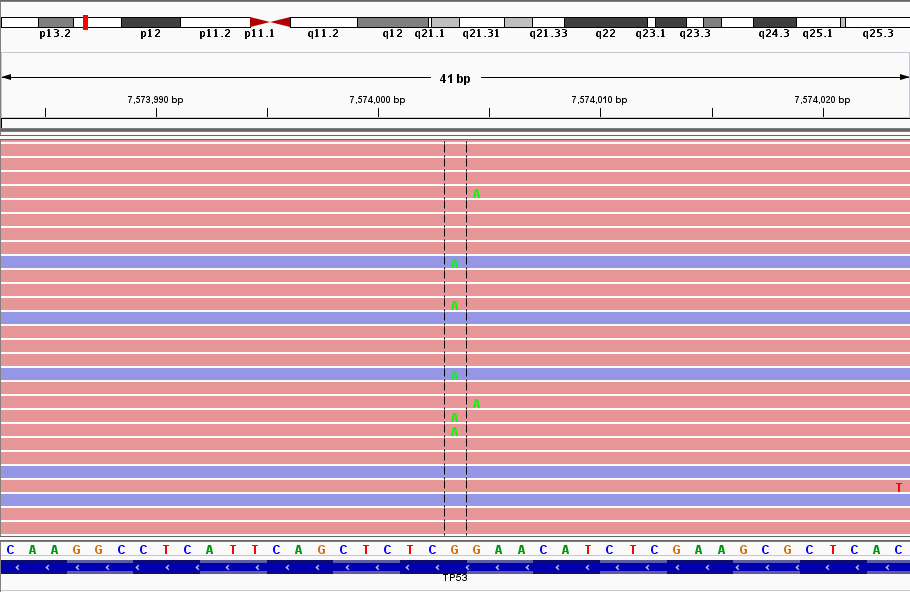


Ion Torrent

sense


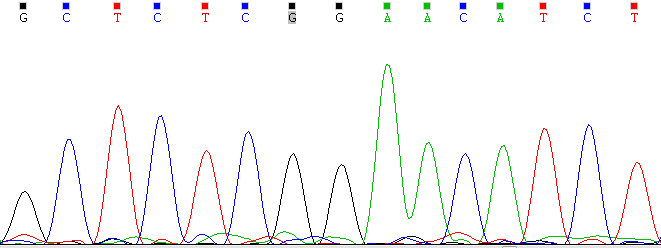


Sanger

#3

antisense

s
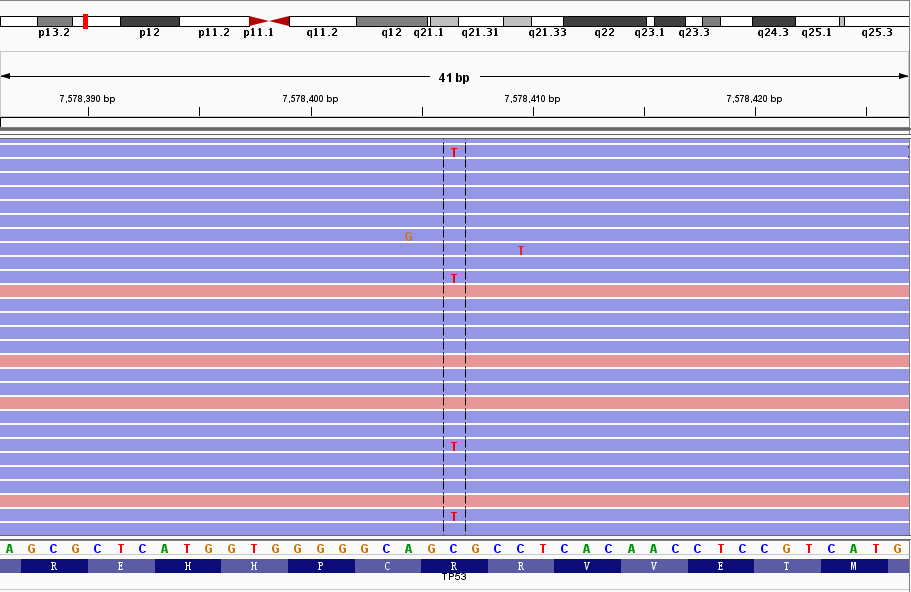


Ion Torrent


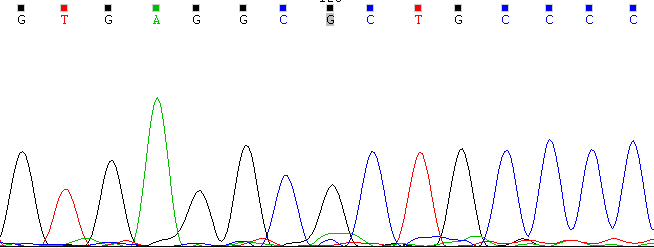


Sanger

Ion Torrent
